# Sweden surpasses the UNAIDS 95-95-95 target: estimating HIV-1 incidence, 2003 to 2022

**DOI:** 10.2807/1560-7917.ES.2024.29.42.2400058

**Published:** 2024-10-17

**Authors:** Erik Lundgren, Macauley Locke, Ethan Romero-Severson, Mira Dimitrijevic, Maria Axelsson, Emmi Andersson, Christina Carlander, Johanna Brännström, Hans Norrgren, Fredrik Mansson, Olof Elvstam, Magnus Gisslén, Lisa Fohlin, Anders Sönnerborg, Jan Albert, Thomas Leitner

**Affiliations:** 1 Theoretical Biology & Biophysics Group, Los Alamos National Laboratory, Los Alamos, New Mexico, United States; 2 Public Health Agency of Sweden, Solna, Sweden; 3 Department of Medicine Huddinge, Karolinska Institutet, Stockholm, Sweden; 4 Department of Infectious Diseases, Karolinska University Hospital, Stockholm, Sweden; 5 Department of Medical Epidemiology and Biostatistics, Karolinska Institutet, Stockholm, Sweden; 6 Department of Infectious Diseases, Södersjukhuset, Stockholm, Sweden; 7 Department of Clinical Science and Education (KISÖS), Karolinska Institutet, Stockholm, Sweden; 8 Department of Clinical Sciences Lund, Division of Infection Medicine, Lund University, Lund, Sweden; 9 Department of Translational Medicine, Clinical Infection Medicine, Lund University, Malmö, Sweden; 10 Department of Infectious Diseases, Växjö Central Hospital, Växjö, Sweden; 11 Department of Infectious Diseases, Institute of Biomedicine, Sahlgrenska Academy, University of Gothenburg, Gothenburg, Sweden; 12 Region Västra Götaland, Department of Infectious Diseases, Sahlgrenska University Hospital, Gothenburg, Sweden; 13 Region Jämtland Härjedalen, Östersund, Sweden; 14 Department of Microbiology, Tumor and Cell Biology, Karolinska Institutet, Stockholm, Sweden; 15 Department of Clinical Microbiology, Karolinska University Hospital, Stockholm, Sweden; * These authors contributed equally to this work and share first authorship.

**Keywords:** HIV, incidence, UNAIDS, biomarkers, modelling

## Abstract

**Background:**

Sweden reached the UNAIDS 90–90–90 target in 2015. It is important to reassess the HIV epidemiological situation due to ever-changing migration patterns, the roll-out of PrEP and the impact of the COVID-19 pandemic.

**Aim:**

We aimed to assess the progress towards the UNAIDS 95–95–95 targets in Sweden by estimating the proportion of undiagnosed people with HIV (PWHIV) and HIV incidence trends.

**Methods:**

We used routine laboratory data to inform a biomarker model of time since infection. When available, we used previous negative test dates, arrival dates for PWHIV from abroad and transmission modes to inform our incidence model. We also used data collected from the Swedish InfCareHIV register on antiretroviral therapy (ART).

**Results:**

The yearly incidence of HIV in Sweden decreased after 2014. In part, this was because the fraction of undiagnosed PWHIV had decreased almost twofold since 2006. After 2015, three of four PWHIV in Sweden were diagnosed within 1.9 and 3.2 years after infection among men who have sex with men and in heterosexual groups, respectively. While 80% of new PWHIV in Sweden acquired HIV before immigration, they make up 50% of the current PWHIV in Sweden. By 2022, 96% of all PWHIV in Sweden had been diagnosed, and 99% of them were on ART, with 98% virally suppressed.

**Conclusions:**

By 2022, about half of all PWHIV in Sweden acquired HIV abroad. Using our new biomarker model, we assess that Sweden has reached the UNAIDS goal at 96–99–98.

**Key public health message**

**What did you want to address in this study and why?**

We wanted to estimate recent HIV incidence trends in Sweden by including undiagnosed people living with HIV (PWHIV) and to assess progress towards the UNAIDS 95–95–95 targets (95% are diagnosed, 95% of them on treatment and in 95% of those, treatment is effective). With migration of PWHIV from abroad, the roll-out of prevention medication and the impact of the COVID-19 pandemic, it is unclear how the HIV situation has changed in recent years.

**What have we learnt from this study?**

Using a new mathematical model that estimates the actual time since infection for diagnosed PWHIV, we found that the yearly number of new HIV acquisitions in Sweden decreased after 2014, with a possible increase in 2021 and 2022. Similarly, the fraction of undiagnosed PWHIV has halved since 2006. For PWHIV in Sweden diagnosed after 2014, 75% were diagnosed within 1.9 for men who have sex with men and within 3.2 years for heterosexual groups.

**What are the implications of your findings for public health?**

Estimating the numbers of undiagnosed PWHIV is at the heart of public health monitoring. By 2022, about half of all PWHIV in Sweden had acquired HIV abroad and half domestically. Nevertheless, Sweden has surpassed the UNAIDS 95–95–95 targets at 96% of all PWHIV being diagnosed, of whom 99% are on antiviral treatment, with 98% being virally suppressed.

## Introduction

Since the detection of the first AIDS cases in 1981, caused by the human immunodeficiency virus (HIV), there have been an estimated 85.6 million infections and there were 39.0 million people with HIV (PWHIV), either HIV-1 or HIV-2, at the end of 2022 [1]. With access to antiretroviral therapy (ART), life expectancy of PWHIV is approaching that of people without HIV [2].

Anonymous and free HIV testing and care is available for anyone in Sweden; free ART has been available since 2004. Testing for HIV is offered to all pregnant women and to migrants. It is also recommended to test people diagnosed with an HIV indicator disease [3]. Most new infections are acquired from undiagnosed PWHIV who are unaware of their HIV status. This means they cannot take preventative measures to reduce onward transmissions, including suppressive ART which is known to prevent further spread [4]. Thus, from a public health perspective, knowledge of the number of undiagnosed PWHIV, along with their demographic data and modes of transmission, is important for HIV prevention [5]. In 2021, the Joint United Nations Programme on HIV and AIDS (UNAIDS) upgraded their original 90–90–90 targets set in 2014 to the 95–95–95 targets. These targets state that by 2025: (i) 95% of all PWHIV should be diagnosed, (ii) 95% of all diagnosed PWHIV should be on ART, and (iii) 95% of those on ART should be virally suppressed [6].

To determine a country’s progress towards the first UNAIDS target (to diagnose at least 95% of PWHIV), it is essential to estimate the total incidence and prevalence of HIV in order to account for PWHIV who still are undiagnosed. This is not a trivial task, owing to several factors such as group differences in risk awareness, unknown time of infection (TI), systematic variation in TI among transmission modes and over calendar years, rate of disease progression, and migration of PWHIV from abroad. Several statistical methods have been developed that use biomarker data (quantifiable molecular data that change predictably over time) to directly estimate the TI of diagnosed PWHIV and subsequently estimate the HIV incidence and the number of undiagnosed PWHIV per year [7-10]. These methods take into consideration the individual’s transmission mode and other demographic information to account for behavioural differences. Other methods, adapted to the unique availability of data and demographic information, have extrapolated the number of PWHIV using a wide range of different techniques, such as back-calculation [11-13].

This study aimed to estimate the annual HIV incidence and fraction of undiagnosed PWHIV in Sweden between 2003 and 2022, using biomarker data available in InfCareHIV, a national quality registry and research database [14]. We focused on HIV-1 because HIV-2 infections account for only < 0.5% of the total number of PWHIV in Sweden [15] and used this information to assess Sweden’s progress towards the 95–95–95 UNAIDS target. Furthermore, we aimed to determine which acquisition modes and demographic characteristics contribute most undiagnosed PWHIV and where efforts therefore need to be focused to surpass the 95–95–95 target.

## Methods

### Data

The data in this study came from the national quality registry and research database InfCareHIV [14], which has a high coverage of clinical measurements and metadata on more than 99% of PWHIV diagnosed in Sweden since 2003, as well as incomplete data going back to 1979; in Supplementary Table S1, we append a detailed description of the demographic data of the InfCareHIV cohort. In total, 13,281 PWHIV were registered in InfCareHIV between 1979 and 2023, and 8,524 PWHIV were still under active care at the time of data extraction on 28 March 2023. The data used in this report included routine laboratory biomarkers such as CD4^+^ T-cell count, HIV-1 viral load and *pol* polymorphism counts derived from genotypic HIV resistance tests. In addition, we used demographic data such as country of birth, estimated country of HIV infection, transmission mode, date of last negative HIV test, date of first positive HIV test in Sweden and, for foreign-born individuals, date of first arrival into Sweden. Information on ART start date, HIV suppression (defined as HIV-RNA < 200 copies/mL), and individuals who have left the InfCareHIV cohort (e.g. deceased, emigrated or lost to follow-up) were also obtained from InfCareHIV. If there was no documented date of the first positive serological HIV test in Sweden, we used the earliest date of a positive biomarker result, including HIV-1 RNA quantification (if after the date of arrival to Sweden). Of the 8,033 foreign-born individuals in the database, 4,644 (58%) had a recorded arrival date to Sweden, with 2,922 (63%) diagnosed after arrival.

### Modelling approach

While the number of PWHIV on ART and the proportion of those virologically suppressed can be obtained directly from InfCareHIV, the first UNAIDS 95 target (fraction diagnosed among all PWHIV) requires statistical estimation of the yearly HIV incidence, which makes it necessary to estimate (i) the actual dates when PWHIV were infected rather than when they were found HIV-positive (diagnosis), and (ii) the number of PWHIV who are not yet diagnosed.

Using a Bayesian approach, we estimated the time of HIV infection for PWHIV in Sweden using a modified multi-biomarker model [8-10]. Here, we used CD4^+^ T-cell count and *pol* polymorphism count for each diagnosed PWHIV, adding multiple measurements (e.g. at follow-up visits) of each biomarker when available, before ART initiation. We also used previous negative test results when available and date of entering Sweden for migrants. The advantage of this biomarker approach is that for all diagnosed PWHIV, we get a probabilistic estimate of the TI, which is usually unknown.

Our incidence estimator adjusted for the undercounting of PWHIV who were infected recently but had not yet been diagnosed and were therefore not recorded in any public health system. This adjustment, based on the inverse probability weighting method proposed in [7], leads to increased uncertainty closer to the present, and that uncertainty declines over time as previously undiagnosed people are diagnosed and recorded. The unit for incidence is number of new cases per year and prevalence is number of PWHIV alive in a given year.

The number of undiagnosed PWHIV in a year is the difference in the prevalence (the cumulative incidence minus all removed PWHIV up to and including that year) and the cumulative number of diagnosed (again minus all removed PWHIV). Here, removed includes PWHIV lost to death or migration out of Sweden. We propagated all uncertainties from the multi-biomarker model and the estimation of the number of undiagnosed by standard Monte Carlo methods up to the final incidence estimates presented in this report.

A large fraction of PWHIV have migrated to Sweden with an already known infection or were diagnosed in Sweden after entry, whereas others have acquired HIV after immigration. We introduced the terms ‘exogenous’ and ‘endogenous’ acquisition to clarify the difference between a person who had acquired HIV before their first arrival to Sweden and a person who acquired HIV after arrival (or was born) in Sweden. We made this distinction to separate acquisitions that occurred in Sweden and were therefore preventable from those that occurred before a person first arrived in Sweden. Note that our method does not assume that all infections in foreign-born persons are exogenous, rather, we account for the uncertainty in both arrival times and TI to determine the probability that acquisition occurred either before or after arrival in Sweden.

A more complete technical specification of the method is appended in the Supplement.

## Results

### The incidence of HIV-1 in Sweden decreased between 2003 and 2022

**Figure 1:**
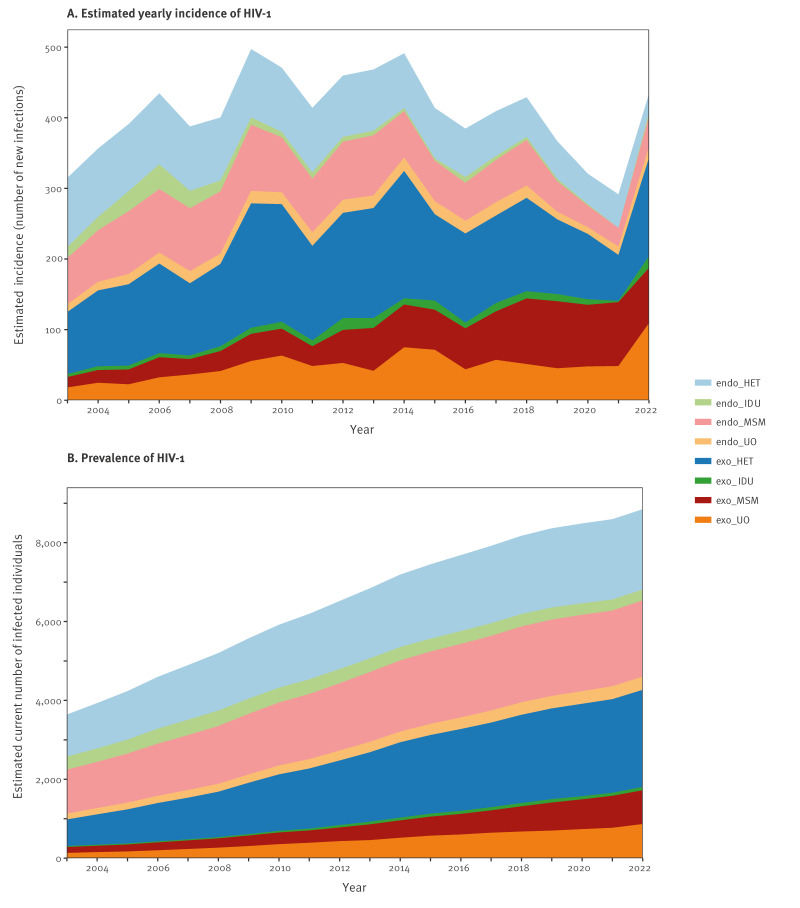
Estimated yearly incidence and current number of infected individuals, by mode of transmission, Sweden, 2003–2022

Figure 1A illustrates the estimated yearly incidence of HIV-1 in Sweden from 2003 until 2022, broken down into transmission categories: endogenous or exogenous infection and transmission modes: transmission among men who have sex with men (MSM), transmission among people who identify as heterosexuals (HET), transmission via injecting drug use (IDU), and unknown/other transmission modes (UO).

Endo: endogenous; exo: exogenous; HET: transmission among people who identify as heterosexuals; MSM: transmission among men who have sex with men; IDU: transmission through injecting drug use; UO: unknown/other transmission modes.

Transmission categories consist of exogenous (outside of Sweden) or endogenous (within Sweden) infections and transmission mode.

Between 2003 and 2009, the estimated yearly incidence of HIV-1 increased; in this period, the largest contribution came from exogenous HET infections. From 2009 and onwards, people with exogenous infections constituted more than half of all new PWHIV in Sweden; in 2009, exogenous HET infections were the largest contributor at 35.5% (95% CrI: 33.1–38.0) of all new infections. With 499 HIV acquisitions, 2009 also had the highest estimated incidence in any year. From 2009 to 2014, the estimated overall HIV-1 incidence fluctuated between 497 (95% CrI: 471–525) and 414 (95% CrI: 388–440) new PWHIV in Sweden yearly, still dominated by exogenous HET infections. After 2014, the overall incidence declined for all the major transmission modes, especially from 2018 onwards.

Acquisition of HIV through heterosexual sex (endogenous and exogenous together) dominated the incidence, ranging from 59.0% in 2003 to 38.6% in 2021. After 2021, it is unclear whether HET continued to dominate because of the larger uncertainty in the model estimates in recent years. In 2021, exogenous acquisitions in MSM made up the largest single transmission category (31.1%; 95% CrI: 27.4–35.1). In 2022, exogeneous HIV acquisition accounted for 79.3% (95% CrI: 69.5–86.6) of all new cases found in Sweden, while endogenous acquisitions decreased from 60.3% (95% CrI: 55.7–65.0) in 2003 to 20.7% (95% CrI: 13.4–30.5) in 2022. 

The largest reduction in any transmission category since 2018 was observed in exogenous HET infections, reducing the number from 132 (95% CrI: 123–143) in 2018 to 65 (95% CrI: 56–77) (twofold) in 2021. In 2022, however, this category increased to 139 (95% CrI: 122–166) new acquisitions (twofold).

Because ART allows PWHIV to survive with an almost average life expectancy, the prevalence of PWHIV was increasing in all transmission categories in the study period (Figure 1B). However, endogenous acquisitions increased more slowly in 2022 than exogenous acquisitions; Prevalent endogenous acquisitions increased by 72.4% from 2,649 (95% CrI: 2,609–2,689) in 2003 to 4,567 (95% CrI: 4,503–4,640) in 2022, while prevalent exogenous acquisitions increased by 339% from 968 (95% CrI: 929–1,008) to 4,253 (95% CrI: 4,198–4,310) within the same period. By 2022, exogenous HET acquisitions accounted for 27.8% (95% CrI: 27.3–28.3) of all prevalent PWHIV. The IDU and UO transmission modes were rare (1,556; 95% CrI: 1,528–1,597), together comprising 17.6% (95% CrI: 17.4–18.0) of the total number of prevalent PWHIV. However, exogenous UO transmission modes contributed 861 (95% CrI: 838–889) PWHIV, constituting 9.8% (95% CrI: 9.5–10.1) of the total HIV-1 prevalence in Sweden. Note that UO cases may be reclassified into one of the defined categories at some later follow-up with the healthcare system, thus reducing the apparent increase of UO cases later on.

### The proportion of undiagnosed PWHIV has decreased between 2003 and 2022

**Figure 2:**
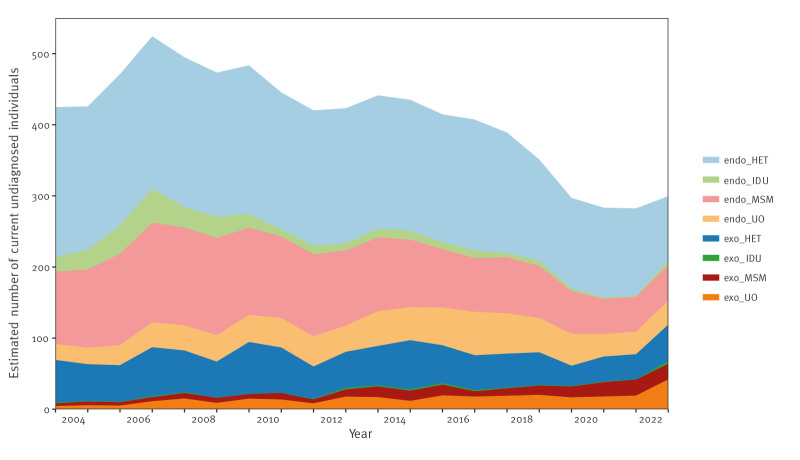
Yearly estimated number of undiagnosed PWHIV in Sweden between 2003 and 2022

To prioritise prevention resources, it is important to estimate the number of undiagnosed PWHIV in different transmission categories. Figure 2 illustrates the estimated number of current undiagnosed PWHIV. From 2003 to 2022, the endogenous HET category declined from 49.5% (95% CrI: 46.1–52.9) to 30.3% (95% CrI: 21.4–41.1) of all undiagnosed PWHIV. Similarly, endogenous HIV acquisitions in the MSM group declined from 24.0% (95% CrI: 21.6–26.4) to 16.8% (95% CrI: 10.9–23.7%). Both the endogenous and exogenous IDU categories were consistently small, and the category endogenous IDU even decreased over time.

Endo: endogenous; exo: exogenous; HET: transmission among people who identify as heterosexuals; MSM: transmission among men who have sex with men; IDU: transmission through injecting drug use; UO: unknown/other transmission modes.

While the estimated number of undiagnosed PWHIV decreased in most transmission categories, it increased by 70.7% in the exogenous HET and UO categories, from 54.6 (95% CrI: 40–72) in 2021 to 93.3 (95% CrI: 65–129) in 2022.

**Figure 3:**
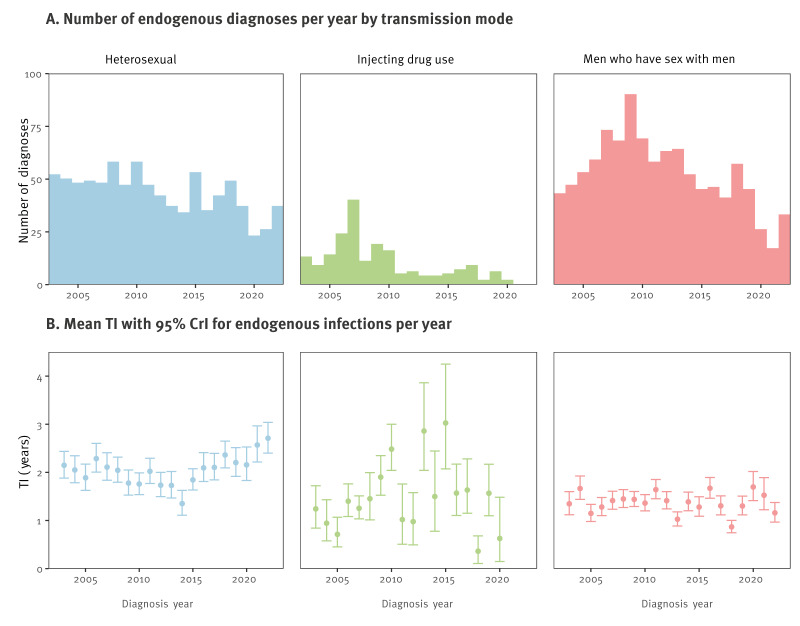
Number of endogenous actual diagnoses and the estimated mean time from infection per year, Sweden, 2003–2022 (n = 2,117)

CrI: credible interval; TI: time from infection to diagnosis.

For endogenous acquisitions, actual diagnoses in all transmission categories decreased during the study period (Figure 3). Yearly endogenous diagnoses in the HET group declined steadily from 52 cases in 2003 to 37 in 2022. While diagnoses in MSM increased between 2003 and 2009, they declined thereafter. The estimated mean TI varied between transmission modes. For HET transmission, the mean TI increased from 2 years to 2.7 years after 2015 (p = 0.027, Mann–Whitney U-test). For transmission related to IDU, the mean TI fluctuated between 0.36 and 3.03, which can be explained by the low numbers of PWHIV in this transmission category. The TI among MSM remained relatively stable from 2003 to 2022, fluctuating between 0.87 and 1.70 years. For infections related to HET transmission, the number of PWHIV in the advanced clinical stage at the time of diagnosis remained fairly constant, while among MSM it declined after 2012; we refer to Supplementary Figure S3 for a display of the numbers of diagnoses in the advanced clinical stage per transmission group. From a modelling point of view, the clinical stage at time of diagnosis does not affect the estimation of incidence. It is, however, interesting that the longer TI correlated with the number of diagnoses in the advanced clinical stage.

### Sweden reached the UNAIDS 95–95–95 target in 2018

**Figure 4:**
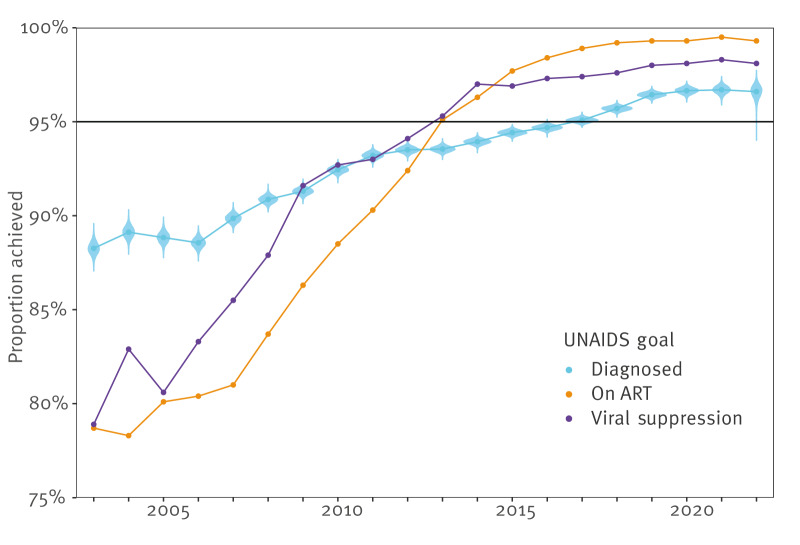
Progress towards the UNAIDS 95–95–95 goals in Sweden, 2003–2022

ART: antiretroviral therapy; UNAIDS: Joint United Nations Programme on HIV/AIDS.

Figure 4 shows that Sweden in 2013 reached the UNAIDS 95% targets for proportions of PWHIV on ART and those with successful viral suppression. The 95% proportion of diagnosed was reached in 2018 (Figure 4). The proportion of PWHIV diagnosed per year has been above 95% for all exogenous transmission modes since 2010; in Supplementary Figure S1 we provide details about diagnoses in individual transmission modes. Reaching 95% diagnoses among endogenous acquisitions was more difficult, especially in the HET category, and occurred only in 2022. For endogenous MSM transmissions, the target 95% diagnosed was reached in 2014, and for IDU transmission, it was reached already in 2009. Across all transmission categories in 2022, diagnoses of endogenous acquisitions were at 96.0% (95% CrI: 94.8–97.0) and exogenous acquisitions at 97.2% (95% CrI: 96.3–97.9).

The first goal, fraction diagnosed among all infected, is shown as a violin plot (where the whiskers indicate the 95% credible interval) estimated by our biomarker-informed incidence model. The other goals are real fractions informed directly from Sweden’s National Quality Registry InfCareHIV.

## Discussion

We have shown that Sweden had reached each of the three UNAIDS 95–95–95 targets by 2022, with 96% PWHIV being diagnosed, 99% of diagnosed being on ART, and 98% of those on ART being virally suppressed.

To estimate the first of the three 95–95–95 targets, the fraction of diagnosed PWHIV among all PWHIV (diagnosed and undiagnosed), we developed a new biomarker-informed incidence estimator. We used time-continuous biomarker trends to estimate TI which was described by a posterior probability distribution of possible infection times, thus moving the incidence of diagnosed PWHIV probabilistically back in time to when the person acquired the infection. To estimate the number of undiagnosed PWHIV in a year, we used the probability of being diagnosed in the years after the acquisition for each transmission mode. To estimate TI, our model can use several biomarkers [6,8] and several measurements of each biomarker from a diagnosed PWHIV, e.g. CD4^+^ T-cell counts and sequencing results, as well as last negative and first positive HIV test results. In addition, for incidence, it also considers start of ART and arrival date for people who migrated to Sweden. One advantage of this method is that the final incidence estimate integrated over both uncertainties in the biomarker-based TI and the estimation of undiagnosed PWHIV to report the confidence in our yearly estimates properly.

The distinction between endogenous and exogenous acquisitions [16] is meant to describe the difference between a person who was born in Sweden or acquired HIV after arrival in Sweden and a person who had acquired HIV before first arrival to Sweden. However, some potential uncertainty may introduce limitations in interpretation: For PWHIV in Sweden, it is possible that they acquired HIV abroad while on travel, or that an HIV-positive person from abroad visited them. From a prevention standpoint, these are preventable infections that occurred in a person living in Sweden, whom Swedish prevention efforts could have reached. These are thus included among endogenous acquisitions. For foreign-born persons, it only matters where the acquisition took place, in Sweden or abroad before arriving in Sweden, to assign the acquisition as endogenous or exogenous. Furthermore, when the estimated TI included the time of arrival to Sweden, the incidence was probabilistically split between endogenous and exogenous acquisitions. Other limitations relate to size and quality of the available data. In our case, some individuals lacked exact data on immigration date or biomarkers, for whom we calculated typical distribution for the missing information. While this procedure made it possible to estimate incidence for the entire population, it introduced additional uncertainties. 

While HIV-1 outbreaks among people who inject drugs (PWID) have occurred in Sweden [17], the overall burden of IDU-associated acquisitions has been low. Endogenous IDU acquisitions have decreased since the early 2010s, after needle exchange programmes became legal in Sweden in 2006. Increased testing for HIV in PWID in healthcare settings, rehab institutions and prisons has also contributed to the low incidence and prevalence of HIV among PWID. In addition, most PWID are efficiently treated with antiretrovirals, and this could also have contributed to a lower incidence in this group.

After 2018, there was a large decrease in HIV incidence that lasted until 2021. The main drivers of this decrease involved endogenous acquisitions among MSM and exogenous HET acquisitions, which both halved their incidence (from 64 to 26 cases and 132 to 65 cases, respectively). This decrease coincided with the recommendation in 2014 to treat all PWHIV regardless of CD4^+^ T-cell count [18], known as TasP (Treatment as Prevention), and with the gradual roll-out of PrEP among Swedish MSM that started in 2018 [18-20]. In addition, it is likely that fluctuations in migration and travel, as well as the many social and medical responses to the COVID-19 pandemic, had effects on the HIV epidemic; physical distancing led to fewer interactions and hence lower risk for sexual acquisitions [21]. While endogenous acquisitions in the HET and MSM categories were already decreasing in the years before the pandemic, physical distancing probably enhanced that trend in the period 2019 to 2021. Meanwhile, exogenous HET acquisitions decreased more drastically during those years, probably due to travel and immigration restrictions implemented by many countries during the COVID-19 pandemic. Additional effects stemming from fewer individuals seeking medical care and delayed healthcare for all types of diseases are also likely to have affected HIV incidence trends. The large increase in incidence in 2022 is probably a result of several factors: (i) lifted travel restrictions led to a surge in travel and migration, (ii) refugees seeking safety from the war in Ukraine [22], and (iii) a backlog of medical services restarting.

The large proportion of PWHIV in Sweden who are virally suppressed by ART is an important contributor to breaking the transmission chains and preventing new infections. However, the still undiagnosed PWHIV in the endogenous HET category call for further investigation, so that efficient targeted or general strategies for diagnosis and prevention can be designed. To reach all individuals with undiagnosed infections, awareness of HIV status needs to be raised also beyond the key populations, and integration of HIV testing in diagnostic algorithms at all levels of care improved.

In Sweden, PrEP is currently almost exclusively provided to MSM and transgender individuals but very low in heterosexual and other groups (data not shown). Expanding access to PrEP for migrant groups is important, but HIV prevention for these populations is complex and involves also other measures [23]. It requires addressing cultural, socioeconomic and systemic barriers through targeted community engagement, comprehensive education and culturally competent healthcare services. In addition, it is crucial to ensure a low barrier for HIV testing to facilitate early diagnosis in these groups.

In addition to the three 95–95–95 targets, a fourth target has been proposed [24]: This goal aims to ensure that 95% of PWHIV on ART experience a good health-related quality of life. By integrating quality-of-life measurements into routine care, healthcare providers can ensure a more comprehensive approach to managing PWHIV, as well as a basis for policy making and development of support services.

## Conclusions

Using a new probabilistic biomarker-based incidence estimator and an extensive national quality registry and research database, we estimate that in 2018, Sweden surpassed each of the three UNAIDS 95–95–95 targets. As suggested by our model, most undiagnosed PWHIV had acquired HIV through HET contact while living in Sweden, but undiagnosed individuals were estimated to exist in all categories of sexually acquired HIV infections. Undiagnosed PWHIV who acquired HIV through intravenous drug use were uncommon, according to our estimates. The results point to successful prevention and diagnostic efforts, especially in PWID and in MSM residing in Sweden, and support the continuation of existing targeted preventive measures such as needle exchange programmes and PrEP, which are also co-organised with access to testing.

## Supplementary Data

795000 Supplementary Material
