# Supplementary material for: Sweden surpasses the UNAIDS 95-95-95 target: estimating HIV-1 incidence, 2003 to 2022
Source: Euro Surveill. 2024 Oct 17;29(42):2400058. doi: 10.2807/1560-7917.ES.2024.29.42.2400058 (PMC11487918; doi:10.2807/1560-7917.ES.2024.29.42.2400058)
Supplement: Supplementary Material [file 24-00058_LEITNER_Supplement.pdf]

Supplementary material

This supplementary material is hosted by *Eurosurveillance* as supporting information alongside the article 'Sweden surpasses the UNAIDS 95-95-95 target: estimating HIV-1 incidence, 2003 to 2022', on behalf of the authors, who remain responsible for the accuracy and appropriateness of the content. The same standards for ethics, copyright, attributions and permissions as for the article apply. Supplements are not edited by *Eurosurveillance* and the journal is not responsible for the maintenance of any links or email addresses provided therein.

InfCareHIV

We trained the multi-biomarker model used in this paper in previously published work using 30 treatment-naive people with HIV (PWHIV) with known infection times and longitudinal measurements of biomarkers over the course of infection [6, 8]. Data for the study came from InfCareHIV, a national HIV reporting database used to track and assess care provided to PWHIV in Sweden [14]. As of the 4th of April, 2023, InfCareHIV had recorded 13,281 HIV cases dating back to 1979. There were 8524 PWHIV under active care by the 4th of April, 2023. Relevant to our study, InfCareHIV collects the date of the first positive HIV test, last negative test, viral load counts, CD4 counts, and HIV sequences collected for drug resistance testing. Additional information also includes age, country of birth, suspected country of infection, transmission mode (heterosexual (HET), men-who-have-sex-with-men (MSM), injecting drug use (IDU), and unknown/other (UO)), and clinical information regarding ART. The database also records the dates of removal and reintroduction into the care system. Supplementary Table 1 summarises the demographic information of all patients in InfCareHIV. Two thousand records included no useable biomarker data, while forty-eight records had no useable information. We implemented a sequence of quality control steps, described below, to remove data that were likely influenced by processing and data entry errors.

Quality control of biomarkers.

If an HIV sequence was recorded before a known first positive date, then the date of the first HIV sequence was taken as the diagnosis date. However, if the date of the first HIV sequence was within 10 days of exactly one year before the first known HIV positive data, we assumed it was a simple typo and put the diagnosis date as exactly one year before the first known HIV positive date. In some instances, InfCareHIV recorded a negative test on the same day as the first positive test. We simply removed the same-day negative test from the data in these situations.

For CD4 counts, if a PWHIV had a CD4 count measurement before their quality-controlled first positive test date, we did not use the CD4 measurement in the multi-biomarker model. We also cleaned sequences to remove unrealistic distributions of polymorphic characters based on distributions from carefully curated HIV sequences for the 30 treatment-naïve people discussed

in the previous section. This prevents high "pol" measurements due to poor sequence quality affecting the posterior estimation. Some PWHIV had multiple different sequences recorded on the same date. If those sequences had different counts of polymorphic sites, then we used the value with the highest proportion of polymorphic sites. To minimise the effect of ART on the determination of posterior distributions of infection times, we excluded any CD4, or "pol" measurements taken more than three days after the start of ART. Furthermore, 43 individuals infected with HIV-2 were removed from our analysis.

Classification of endogenous and exogenous PWHIV

We introduced an endogenous and exogenous classification into our model, similar to Giardina et al. [8], to allow us to estimate the number ofPWHIV infected before arrival to Sweden. PWHIV who were infected with HIV-1 whilst they were residents of Sweden, either because they were born there or immigrated, are in the endogenous category. PWHIV infected with HIV before arrival to Sweden are in the exogenous category. PWHIV who were born outside of Sweden without a previous negative HIV test in Sweden are potentially infected with HIV before or after immigrating. These people are *potentially* exogenous, while PWHIV, born in Sweden or have a negative HIV test after arrival, constitute *known* endogenous acquisitions.

Estimation of time since infection distributions

We used the biomarker data after quality control in our multi-biomarker model according to the method previously described by Giardina et al [8] and Lundgren et al. [10] to determine posterior distributions of time since infection (TI). To estimate the incidence in Sweden, we used these distributions. If neither CD4 or polymorphism counts were available, we used "typical" distributions (described in the following section) for TI distributions given endogenous/exogenous categorisation and the mode of transmission.

Typical TI distributions

For individuals who lacked useable biomarker data, we used a "typical" distribution. We considered "typical" distributions for combinations of endogenous/exogenous categorisation and transmission mode (HET, MSM, IDU and U/O). We found these by averaging all informed distributions for individuals within a given combination. That is, the typical distribution for combination C_{tr} is,

$$f^{(C_{tr})}(t) = \frac{1}{n_{C_{tr}}} \sum_{i \in C_{tr}} f_i(t)$$

where $f_i(t)$ is the probability density function (pdf) for the time t between infection and diagnosis of PWHIV i and $n_{C_{tr}}$ is the number of PWHIV in category C_{tr} with informative biomarkers. Here C is the endogenous/exogenous categorisation, and tr is the transmission

mode The U/O transmission mode had few documented PWHIV; the "typical" distribution for this mode was found by pooling all transmission modes and finding the average.

Estimation of unknown arrival times

Within the data we received, some potentially exogenous PWHIV did not have known arrival dates in Sweden. Similarly, to how we handled individuals with no useable biomarkers, we inferred a "typical" distribution for the time between diagnosis and arrival in Sweden. We stratified these distributions by transmission mode to provide an estimate of arrival time for people with no recorded arrival time. We obtained these distributions using the kernel density estimation with the "density" function in "R". It is important to note that, since diagnosis can happen before or after arrival in Sweden, this time can take both positive and negative values.

Estimation of incidence

To estimate the incidence of HIV-1 in Sweden, we used Monte Carlo (MC) sampling to estimate means and distributions of incidence and resulting statistics. In each MC interaction, we drew an infection time independently for each PWHIV by subtracting a random draw from their TA distribution from their diagnosis date. Potentially exogenous cases that did not possess an arrival date had an entry date estimated by using the diagnosis date and a draw from the typical arrival time distribution for the correct transmission categorisation. For known endogenous individuals and potentially exogenous individuals with an infection time later than their arrival time, their contribution to incidence was counted as the year during which the infection occurred. For potentially exogenous individuals with an infection time before the arrival time, we instead counted the contribution as the year during which the individual arrived in Sweden. This provided an estimate of the number of infections each year for the individuals diagnosed; however, to find the incidence, we had to estimate the total number of infections, including those not diagnosed.

To account for infected, but not yet diagnosed people, we computed a sample weight based on the inverse of the probability that a person diagnosed at that time would have been diagnosed by the end of the study period. Inverse probability weighting is a way of estimating the size of a population from which an event with a known probability occurred. Suppose $F^{C_{tr}}$ is the cumulative density function (cdf) of the typical distribution for category C_{tr}. In that case, the probability of an individual infected with HIV-1 at time u_{ij} diagnosed before the study end time τ is $1/F^{C_{tr}}(\tau - u_{ij})$, where u_{ij} is the jth sampled infection time for the ith PWHIV. We could have used the inverse of this probability, $1/F^{C_{tr}}(\tau - u_{ij})$, as the contribution weight for each individual; however, to better reflect the estimate's uncertainty, we used a geometric random variable to modify the weight. For example, if the first trial in the geometric random variable was a “success”, the individual would contribute a value of 1 to the incidence, but if there were two “failures” before a success, the individual would contribute a value of 3 to the incidence. We used success probability $F^{C_{tr}}(\tau - u_{ij})$ such that the expected value is $1/F^{C_{tr}}(\tau - u_{ij})$ as desired.

Estimation of the current cases

While data were not complete before 2003, we assumed that anyone still living in Sweden would be in InfCareHIV by that time, and it was still meaningful to keep track of infections from before that time that we had records of. We then found the cumulative sums of the number of diagnoses and estimated incidence. We subtracted the number of patients who died or left Sweden from these numbers. This resulted in the current number of people diagnosed with HIV and an estimate of the current total number of PWHIV in Sweden. We then found an estimate for the number of undiagnosed cases by subtracting the diagnosed cases from the estimate of the total number of cases. Likewise, we found the diagnosed fraction by dividing the diagnosed number by the total.

Estimation of the mean TI by diagnosis year

We estimated the mean TI for individuals diagnosed in each one-year interval between 2003 and 2022 for the HET, IDU, and MSM transmission modes. Only known endogenous individuals were considered for this part of the analysis to avoid potential biases since the arrival time would cut off the distributions. For each interval and transmission mode combination, we drew one TI from each individual in that category and found the mean of all the drawn TIs. We repeated this procedure to find 10^4 samples of the mean, which we used to find the overall mean and 2.5th and 97.5th percentile values in each category.

Year of HIV diagnosis	<1987	1987-1996	1997-2006	2007-2016	2017-2023	Unknown	All
All<br>(Percent)	963<br>(7.3%)	2512<br>(19%)	2928<br>(22%)	4404<br>(33%)	2423<br>(18%)	51<br>(0.4%)	13281<br>(100%)
**Sex**							
Female<br>(Percent)	114<br>(12%)	643<br>(26%)	1111<br>(38%)	1717<br>(39%)	869<br>(36%)	3<br>(5.9%)	4457<br>(34%)
Male<br>(Percent)	849<br>(88%)	1869<br>(74%)	1817<br>(62%)	2687<br>(61%)	1536<br>(63%)	46<br>(90%)	8804<br>(66%)
Unknown<br>(Percent)	0<br>(0%)	0<br>(0%)	0<br>(0%)	0<br>(0%)	18<br>(0.7%)	2<br>3.9%)	20<br>(0.2%)
**Birth region**							
Sweden<br>(Percent)	629<br>(65%)	1208<br>(48%)	1130<br>(39%)	1138<br>(26%)	450<br>(19%)	12<br>(24%)	4567<br>(34%)
Eastern and southern<br>Africa (Percent)	14<br>(1.5%)	386<br>15%)	553<br>19%)	1016<br>23%)	399<br>16%)	3<br>5.9%)	2371<br>18%)
Western and central<br>Africa (Percent)	2<br>(0.2%)	77<br>(3.1%)	343<br>(12%)	568<br>(13%)	363<br>(15%)	1<br>(2.0%)	1354<br>(10%)
Asia and the Pacific<br>(Percent)	4<br>0.4%)	59<br>2.3%)	304<br>10%)	505<br>11%)	274<br>11%)	3<br>5.9%)	1149<br>8.7%)
Eastern Europe and<br>central Asia (Percent)	0<br>0.0%)	12<br>0.5%)	58<br>2.0%)	190<br>4.3%)	255<br>10.5%)	0<br>0.0%)	515<br>3.9%)
Latin America and<br>the Caribbean (Percent)	15<br>(1.6%)	70<br>(2.8%)	120<br>(4.1%)	194<br>(4.4%)	195<br>(8.0%)	1<br>(2.0%)	595<br>(4.5%)
Middle East and<br>North Africa (Percent)	2<br>0.2%)	84<br>3.3%)	122<br>4.2%)	324<br>7.4%)	150<br>6.2%)	1<br>2.0%)	683<br>5.1%)
Western and central Europe<br>and North America[1] (Percent)	102<br>(11%)	271<br>(11%)	247<br>(8.4%)	410<br>(9.3%)	287<br>(12%)	4<br>(7.8%)	1321<br>(10%)
Unknown<br>(Percent)	195<br>(20%)	345<br>(14%)	51<br>(1.7%)	59<br>(1.3%)	50<br>(2.1%)	26<br>(51%)	726<br>(5.5%)
**Mode of HIV transmission**							
Heterosexual<br>(Percent)	39<br>(4.0%)	945<br>(38%)	1650<br>(56%)	2266<br>(51%)	1051<br>(43%)	3<br>(5.9%)	5954<br>(45%)
Men who have sex with men<br>(Percent)	600<br>(62%)	1011<br>(40%)	773<br>(26%)	1245<br>(28%)	821<br>(34%)	11<br>(22%)	4461<br>(34%)
Intravenous drug use<br>(Percent)	255<br>(26%)	321<br>(13%)	228<br>(7.8%)	217<br>(4.9%)	80<br>(3.3%)	1<br>(2.0%)	1102<br>(8.3%)
Unknown/Other<br>(Percent)	69<br>(7.2%)	235<br>(9.4%)	277<br>(9.5%)	676<br>(15%)	471<br>(19%)	36<br>(71%)	1764<br>(13%)
**Level of immunosuppression**							
Nadir CD4 cell count<br>Median (IQR)	70<br>(13-190)	130<br>(39-240)	191<br>(100-276)	260<br>(140-396)	290<br>(94-490)	NA	220<br>(90-360)
Missing nadir CD4 cell count<br>(Percent)	144<br>(15%)	203<br>(8.1%)	23<br>(0.8%)	26<br>(0.6%)	33<br>(1.4%)	51<br>(100%)	480<br>(3.6%)

Supplementary Table S1. Demographics of the InfCareHIV cohort as of 4 April 2023
Data are numbers and percentages (%) unless otherwise stated. Percentages do not always add up to hundred due to rounding. Birth regions according to UNAIDS definitions. Unknown/other transmission modes combine the following: mother to child, blood products, undefined and missing. NA, not available.

Additional figures

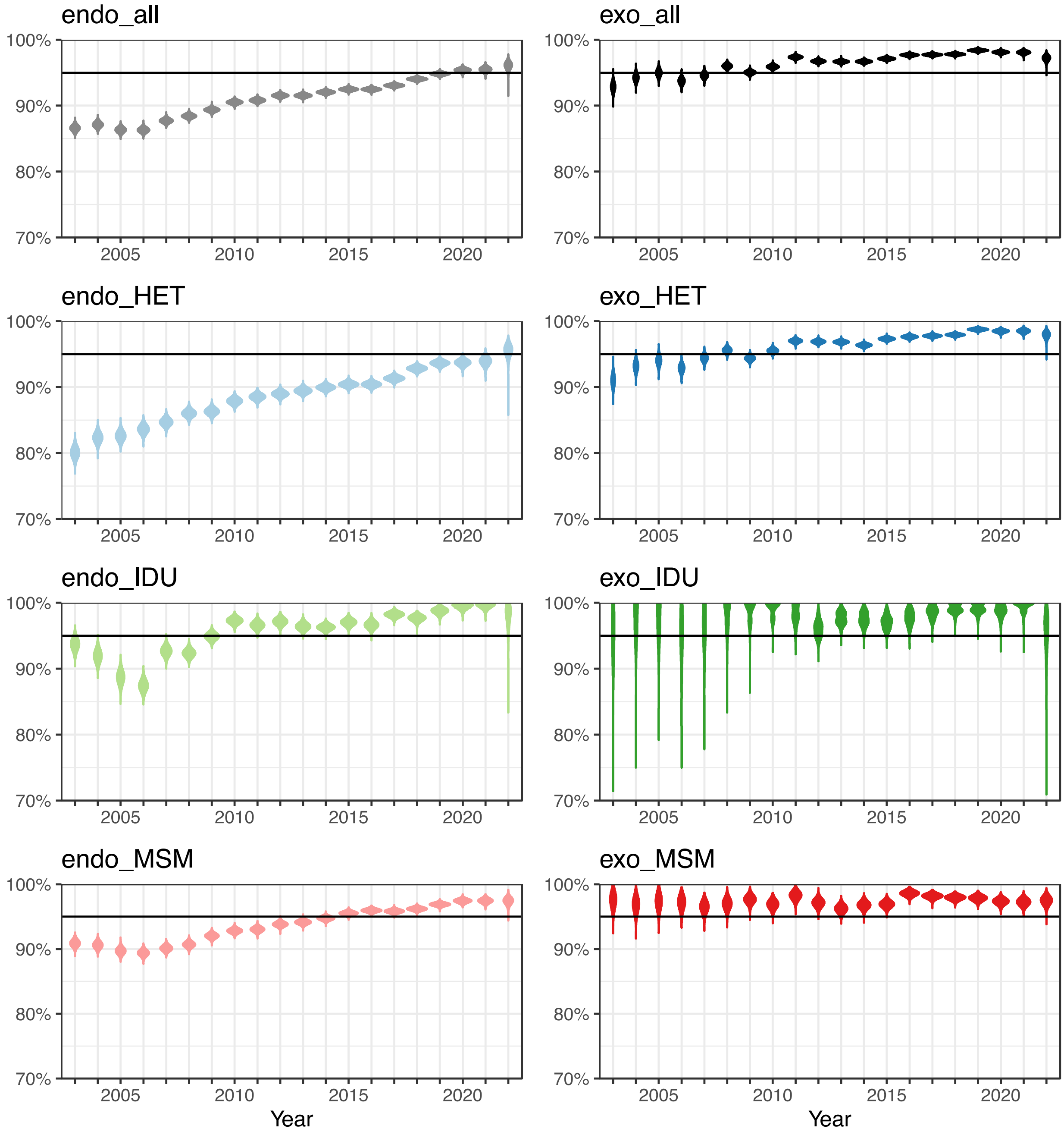

Supplementary Figure S1: Violin plots illustrating for each transmission mode and endogenous/exogenous category, the proportion of diagnosed cases. Black line represents the ``95'' target number from the UNAIDS target. Data presented between 2003-2022.

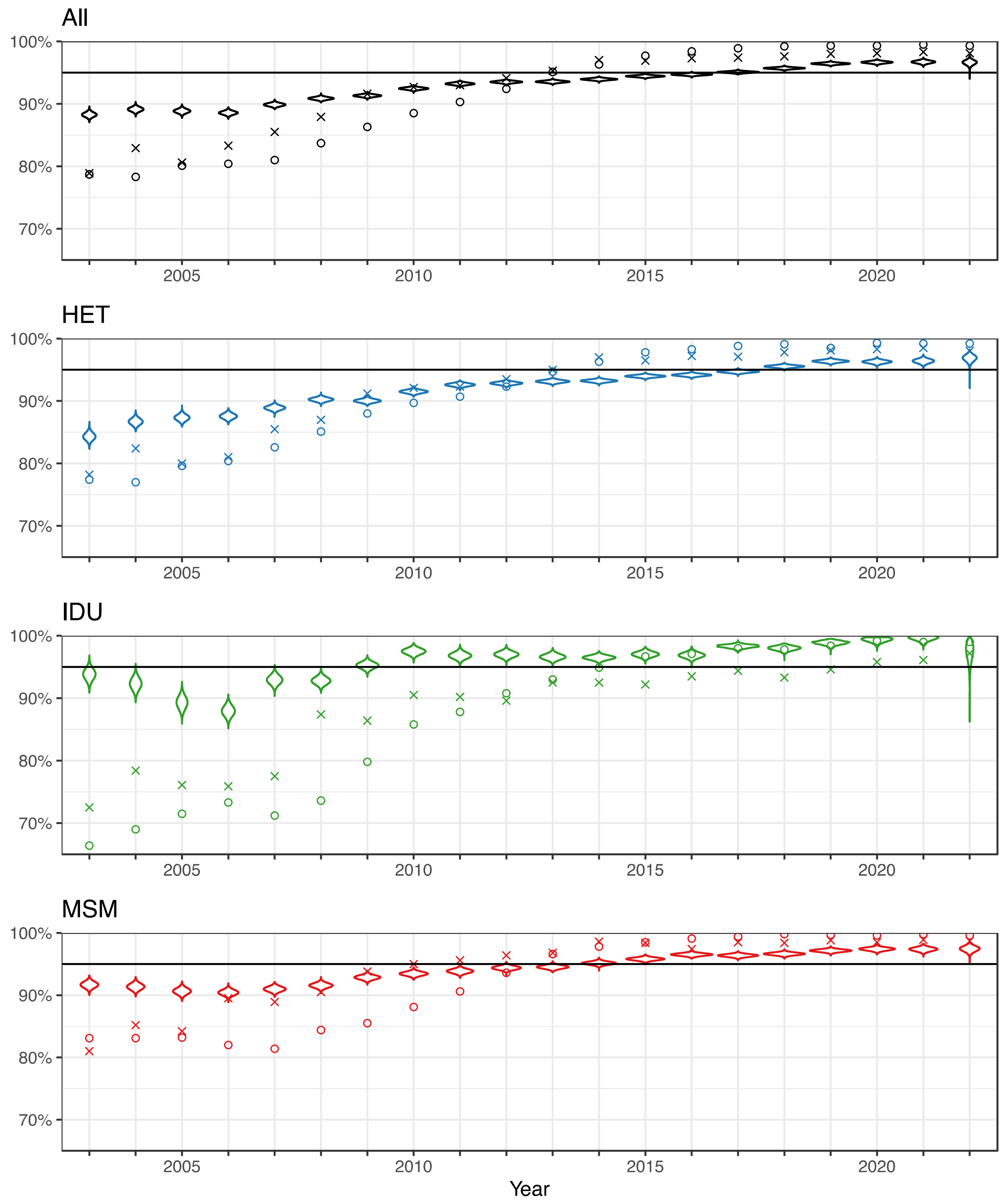

Supplementary Figure S2: Progress for to the UNAIDS 95-95-95 goal broken down by transmission mode, HET (blue), MSM(RED) and IDU(GREEN). Violin plots represent the distribution of the proportion of diagnosed individuals, “circle” proportion of PWHIV on ART, “x” proportion of PWHIV on ART on successful treatment. Black line represents the ``95’’ target number from the UNAIDS target. Data presented between 2003-2022.

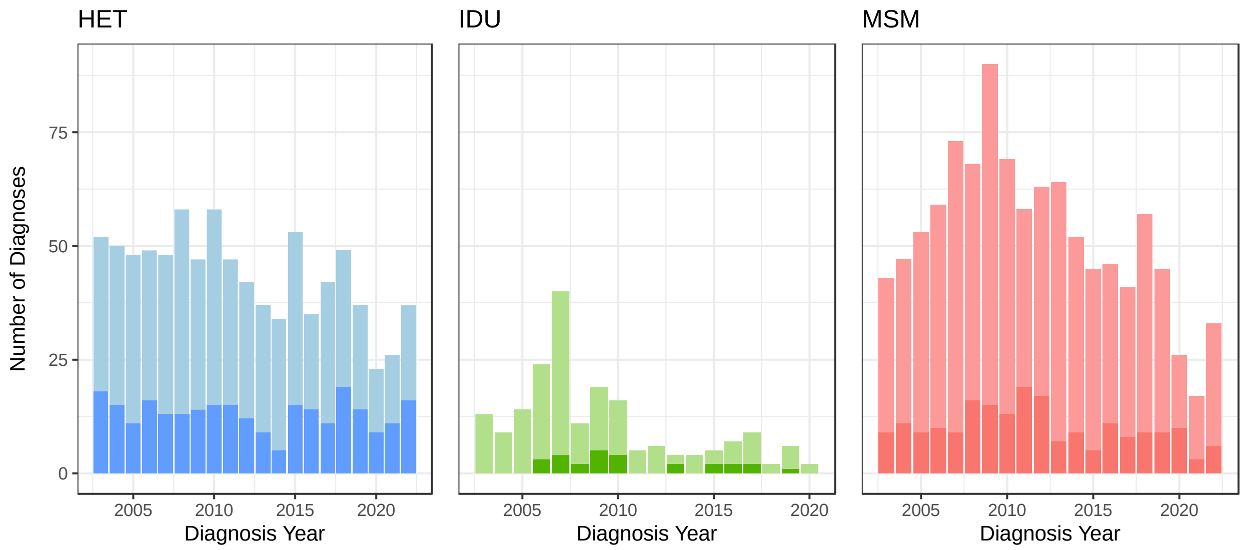

Supplementary Figure S3: Number of endogenous diagnoses per year in Sweden, 2003-2022 (n=2117). We have broken down the number of endogenous diagnoses per year by transmission mode and clinical stage. We defined the clinical stage as advanced (darker region) when a clinician confirmed an AIDS diagnosis within six months of a confirmed HIV-1 diagnosis or, when the first CD4 counts were less than 200 counts within six months of a confirmed HIV-1 diagnosis, or non-advanced (light region) otherwise.
